# Stress-induced reproductive arrest in *Drosophila* occurs through ETH deficiency-mediated suppression of oogenesis and ovulation

**DOI:** 10.1186/s12915-018-0484-9

**Published:** 2018-01-30

**Authors:** Matthew R. Meiselman, Timothy G. Kingan, Michael E. Adams

**Affiliations:** 1 Departments of Entomology and Cell Biology and Neuroscience, University of California, Riverside, CA 92521 USA; 2 Graduate Program in Cell, Molecular and Developmental Biology, University of California, Riverside, CA 92521 USA; 3 Graduate Program in Neuroscience, University of California, Riverside, CA 92521 USA

**Keywords:** Heat and nutrient stress, Endocrine, Liberin, Ecdysone, Ecdysis triggering hormone, Juvenile hormone, Octopamine

## Abstract

**Background:**

Environmental stressors induce changes in endocrine state, leading to energy re-allocation from reproduction to survival. Female *Drosophila melanogaster* respond to thermal and nutrient stressors by arresting egg production through elevation of the steroid hormone ecdysone. However, the mechanisms through which this reproductive arrest occurs are not well understood.

**Results:**

Here we report that stress-induced elevation of ecdysone is accompanied by decreased levels of ecdysis triggering hormone (ETH). Depressed levels of circulating ETH lead to attenuated activity of its targets, including juvenile hormone-producing corpus allatum and, as we describe here for the first time, octopaminergic neurons of the oviduct. Elevation of steroid thereby results in arrested oogenesis, reduced octopaminergic input to the reproductive tract, and consequent suppression of ovulation. ETH mitigates heat or nutritional stress-induced attenuation of fecundity, which suggests that its deficiency is critical to reproductive adaptability.

**Conclusions:**

Our findings indicate that, as a dual regulator of octopamine and juvenile hormone release, ETH provides a link between stress-induced elevation of ecdysone levels and consequent reduction in fecundity.

**Electronic supplementary material:**

The online version of this article (10.1186/s12915-018-0484-9) contains supplementary material, which is available to authorized users.

## Background

Reproduction demands precise temporal and spatial coordination of energy resources in a dynamic environment. Under stressful conditions, organisms make critical metabolic decisions whether to opt for survival at the expense of reproduction or to bear the cost of reproduction at the possible expense of survival [1, 2]. In addition to energetic demands, stressors indicate the environment may not be favorable for maturation of progeny, and animals largely consider either via an adaptive endocrine state. In humans, chronic stress, malnutrition, or excessive exercise, all markers for conditions suboptimal for parturition, suppress fertility by changing endocrine state [3]. This principle is also true for the fruit fly, *Drosophila melanogaster*, which offers opportunities for associating changes in gene expression and endocrine state with phenotypic changes in oogenesis and ovulation.

As arthropods are ectothermic, temperature is a critical factor in evaluating suitability of environmental conditions for procreation. Even brief exposures to cold or heat can deplete energy stores, resulting in attenuation of reproduction [4–6]. Mounting evidence suggests that the adult stress response of *Drosophila* is mediated, at least in part, by ecdysone signaling [7]. Ecdysone receptor mutants are more resistant to heat, starvation, and oxidative stress, and they have a 50% longer lifespan [8], while heat and courtship stress elevate ecdysone levels [9, 10]. In addition to association of elevated steroid levels with reproduction-attenuating stressors [6, 11], artificial elevation of steroid suppresses oogenesis [12, 13], ovulation [14], and egg-laying [15]. Taken together, considerable evidence indicates that ecdysone signaling mediates environmental stress responses, leading to reproductive arrest. However, precise mechanisms through which it exerts this arrest remain undefined.

Since ecdysone facilitates reproductive activities, it is somewhat perplexing that its levels elevate under stressful conditions, thereby inhibiting reproduction. An intriguing explanation lies in the recent revelation that fecundity is critically influenced by ecdysis triggering hormone (ETH) through its actions as an allatotropin [16]. ETH levels in turn are influenced by ecdysone. During the molt, high ecdysone levels induce expression of ETH and ETH receptor (ETHR) genes but suppress Inka cell secretory competence by inhibiting expression of the transcription factor *βFTZ-F1*; ETH release is thereby blocked until ecdysone levels decline [17]. Here we report that ETH signaling in adults is also influenced by steroid levels, with important ramifications for endocrine responses to stress.

Heat stress induces impaired oogenesis, with fewer eggs in the later, vitellogenic stages of synthesis and a greater number of oocytes undergoing programmed cell death [6]. Programmed cell death of nascent oocytes is a well-characterized mechanism for re-allocation of energy stores [18]. Additionally, both heat-stressed and ETH-deficient flies show a paradoxical accumulation of mature oocytes, in spite of diminished oogenesis [16].

Accumulation of mature oocytes is a common phenomenon when local control over ovary and oviduct muscle by paracrine signals including proctolin, glutamate, and octopamine (OA) is impaired [19–22]. Since ETH is a prolific liberin during ecdysis, we asked whether it could modulate release of effectors in the reproductive tract. We report here that disruption of ETH signaling specifically in octopaminergic neurons innervating the female reproductive tract causes anovulation. Moreover, ETH treatment in vitro or in vivo stimulates ovulation.

Using heat and nutritional deprivation as stressors, we show that as circulating levels of ecdysone change, ETH levels do as well, but in an inverse manner. We describe a pattern of reproductive regulation, wherein altered endocrine state adjusts reproductive output in response to stressful environmental conditions.

## Results

### ETH deficiency-associated ovarian egg retention is phenocopied by ETHR knockdown in octopaminergic neurons

We showed previously that ETH deficiency causes increased ovarian retention of mature, stage 14 eggs [16]. In this study, we investigated the cause of this egg retention phenotype and report that octopaminergic neurons innervating ovaries and oviduct are targets of ETH.

**Fig. 1:**
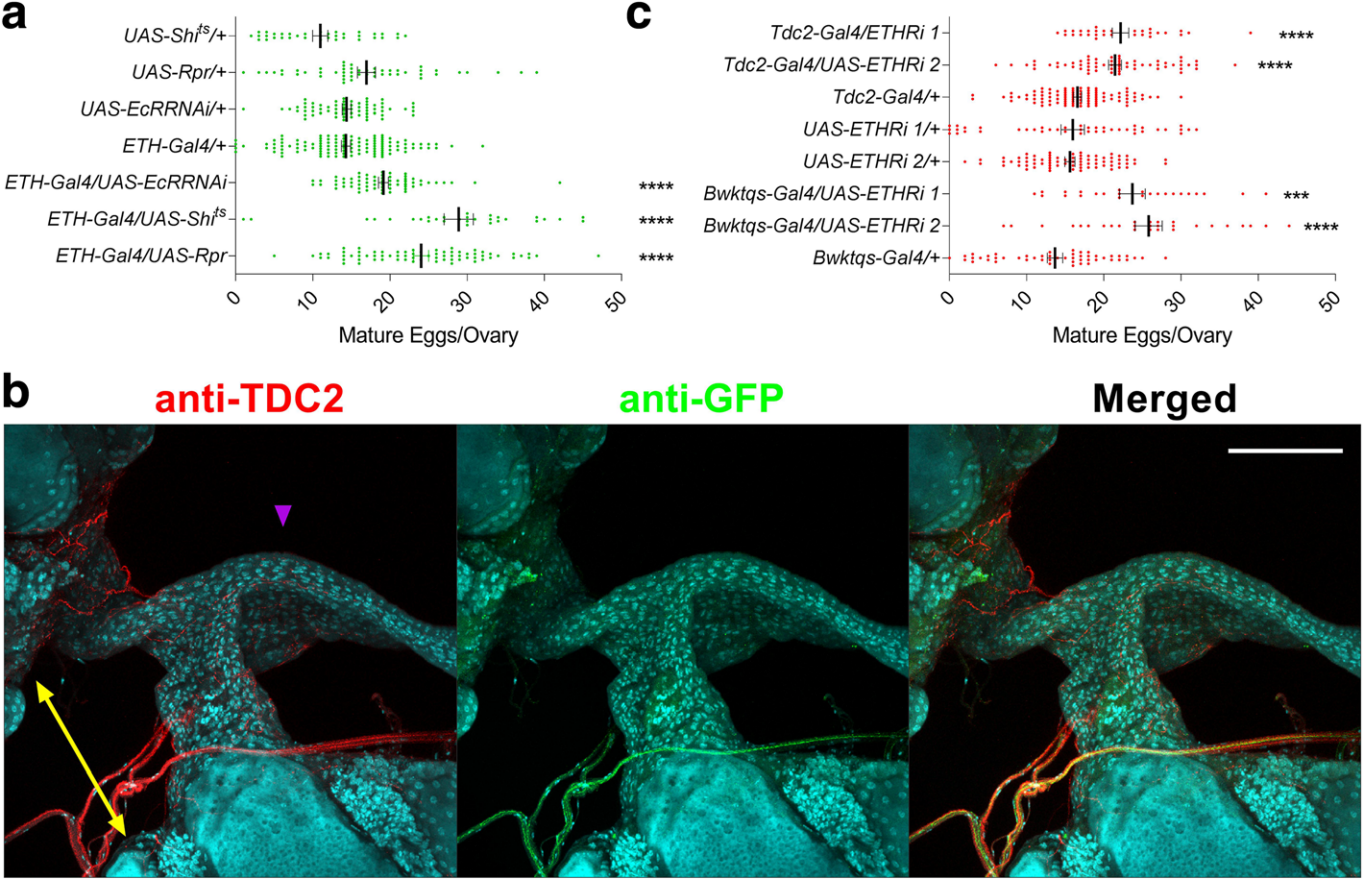
Disruption of ETH action on octopaminergic neurons causes egg retention in virgin females. **a** Number of mature eggs retained in ovaries of isolated adult virgin females with Inka cells ablated (*ETH-Gal4;Tubulin-Gal80*^*ts*^*/UAS-Reaper*) (*n* = 30), blocked (*ETH-Gal4/UAS-Shi*^*ts*^) (*n* = 35), and EcR-silenced (*ETH-Gal4;Tubulin-Gal80*^*ts*^*/UAS-EcR-RNAi*) (*n* = 50) (biological replicates). **b** Co-staining of ETHR positive neurons in the vicinity of the juncture of ovaries (*yellow arrow*) and oviduct (*purple arrowhead*) ETHR-Gal4/UAS-mCD8-GFP (*green*), anti-TDC2 (*red*), and 4’,6-diamidino-2-phenylindole (DAPI, *blue*). Scale bar = 100 μm. **c** Number of mature eggs retained in the ovaries of virgin females with ETHR silenced in all octopaminergic neurons (*Tdc2-Gal4*) (*n* = 50) and in the neurons innervating the female reproductive tract (*Bwk*^*tqs*^*-Gal4*) (*n* = 40) (biological replicates). Error bars represent standard error of the mean (SEM). ***p < 0.001, ****p < 0.0001

To further characterize physiological consequences of ETH deficiency on the reproductive tract, ETH release from Inka cells was disrupted using several experimental approaches. Reduction of ecdysone receptor expression selectively in Inka cells, the sole source of ETH, leads to significant increase in mature egg retention in ovaries of 4-day-old virgin females (Fig. 1a). Likewise, Inka cell ablation or block of ETH release using temperature-sensitive *shibire* expression causes increased egg retention. As expression of either EcR-RNA interference (RNAi) or the *reaper* gene prior to adulthood causes lethal ecdysis deficiencies, we used the temperature-sensitive tubulin-Gal80^ts^ to repress Gal4 and raised flies at the restrictive temperature (18 °C) until after eclosion (see Methods section for details).

To identify cellular targets of ETH responsible for increased egg retention, we used the Trojan ETHR-Gal4 driver for green fluorescent protein (GFP)-mediated visualization and noticed labeling of octopaminergic neurons innervating the base of the ovary and oviduct [23, 24]. These neurons express tyrosine decarboxylase 2 (TDC2), responsible for conversion of tyrosine to tyramine, a critical step in OA synthesis (Fig. 1b) [25]. As OA release and octopamine receptor activation are critical for ovulation in *Drosophila* [21, 26–28] and ETH is known to target neuroendocrine cells [29, 30], we examined whether disruption of ETH signaling in octopaminergic neurons influences ovulation. Indeed, ETHR knockdown in octopaminergic neurons of the female reproductive tract was accomplished using two independent Gal4 drivers: *Tdc2-Gal4*, which drives to all octopaminergic neurons, including those in the female reproductive tract [25], and *Bwk*^*tqs*^*-Gal4*, which labels reproductive tract neurons including octopaminergic cells [22]. ETHR knockdown using either of these drivers increases retention of mature eggs in the ovaries (Fig. 1c).

### ETH induces ovarian contractions and ovulation via activation of octopamine neurons

**Fig. 2:**
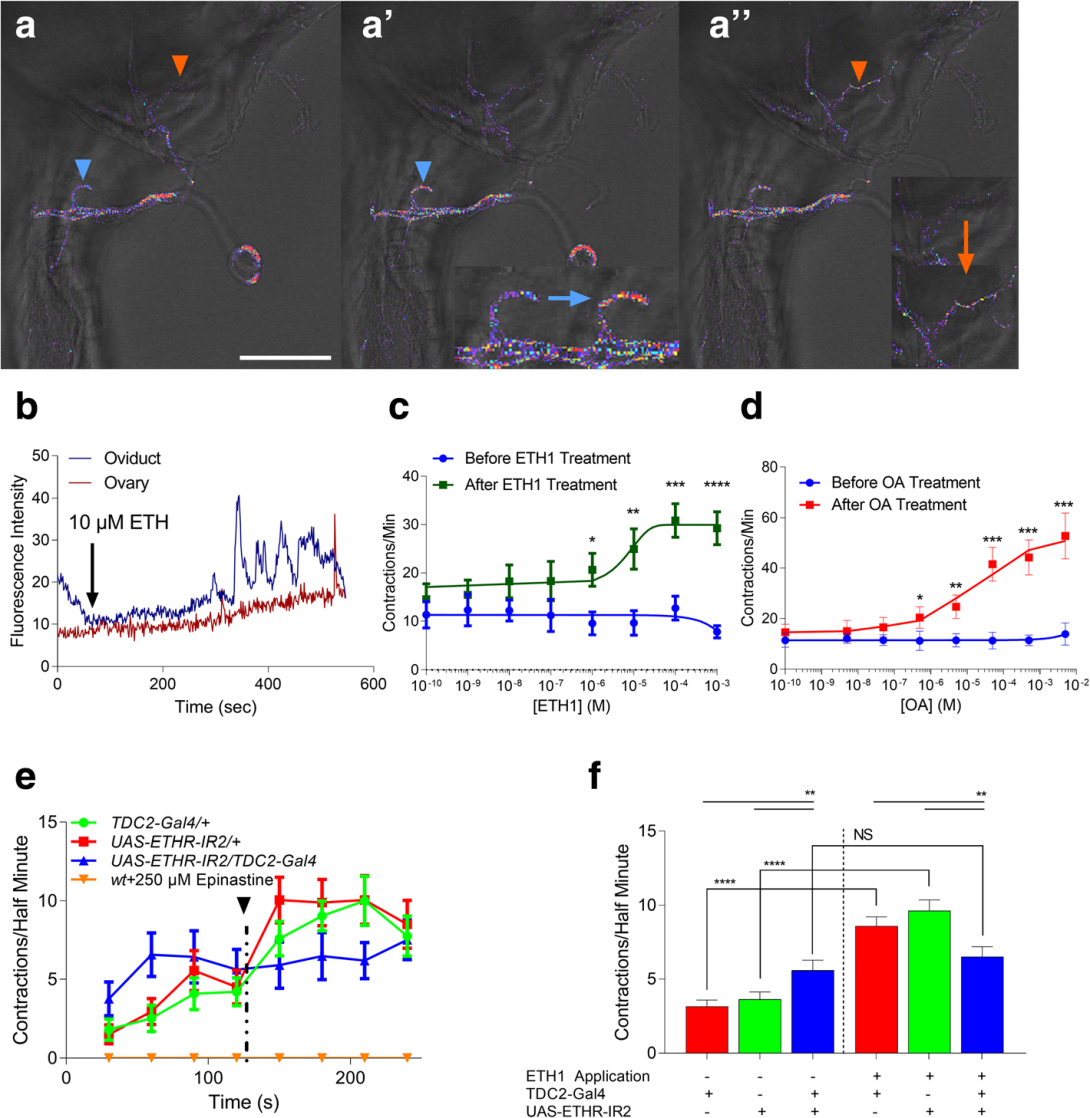
ETH mobilizes calcium in octopaminergic neurons and evokes contractions of the ovarian peritoneal sheath. **a**, **a’**, **a”** Calcium imaging of octopaminergic neurons innervating excised ovary (*red arrowheads*) and oviduct (*blue arrowheads*) of mated females (genotype *Tdc2-Gal4/UAS-GCaMP6S*). Fluorescence responses observed in neurons innervating the oviduct (*blue arrowheads*) before (**a**) and after (**a’** and **a’** inset) ETH1 exposure (10 μM). Change in fluorescence before and after ETH application is indicated by the *horizontal blue arrow*. Fluorescence responses recorded at the base of the ovary (**a”**, *orange arrowhead*) after ETH application (*vertical orange arrow* in **a”** inset); scale bar = 100 μm; insets are 2X **(a’**) and 1.5X (**a”**). **b** Nerve terminal fluorescence intensity from **a** over time, before and after treatment, indicated by *black arrow*. **c**, **d** Contractile responses of ovaries excised from Canton-S females before (*blue*, **c**) and after ETH (*green*, **c**) or octopamine (*red*, **d**) treatment at various concentrations. Statistics indicate difference in contraction frequency between agonist and saline (*n* = 20). **e** Ovarian contractile responses following ETHR silencing in octopaminergic neurons (*TDC2-Gal4/ETHR-IR2*) and genetic controls before and after ETH1 (10 μM) treatment indicated by the *black arrowhead* and *dotted line* (*n* = 25) (biological replicates). **f** Contraction rate of each genotype before and after ETH1 treatment is compared statistically among genotypes or across treatment. Contraction frequency for each genotype was compared both to its contraction frequency before and after ETH treatment (Student’s *t* test) and to other genotypes either before or after ETH treatment (one-way analysis of variance (ANOVA)). Error bars represent SEM. *NS*, *p* > 0.05; *p < 0.05, **p < 0.01, ***p < 0.001, ****p < 0.0001. TDC2-Gal4/+: Red, UAS-ETHR-IR2/+: Green, UAS-ETHR-IR2/TDC2-Gal4: Blue

Since ETH activates ETHR-expressing target cells through calcium elevation [29, 31], we asked whether it mobilizes calcium in octopaminergic neurons innervating the oviduct and ovaries. We exposed the reproductive tract of *Tdc2-Gal4/UAS-GCaMP6S* females to 10 μM ETH in vitro in the presence of the OA receptor antagonist epinastine (10 μM) and Mn^2+^ (50 μM) to suppress movement associated with muscle contractions and observed oscillatory fluorescence responses in OA neurons (Fig. 2a, b); saline controls elicited no fluorescence responses (Additional file 1: Figure S1A, B).

OA is reported to amplify contractions of the peritoneal sheath surrounding the ovaries [23]. We found that exposure of excised ovaries to either OA or ETH leads to contractions of the peritoneal sheath in a concentration-dependent fashion (Fig. 2c, d, Additional file 2: Video S1 and Additional file 3: Video S2). Additionally, silencing ETHR expression in OA neurons eliminates contractile responses to 10 μM ETH (Fig. 2e, f). Interestingly, in addition to the expected decrease in post-treatment contraction frequency, constitutive, pre-treatment contraction frequency of the peritoneal sheath increases significantly in flies following ETHR silencing in octopaminergic neurons (*TDC2-Gal4 > UAS-ETHR-IR2*) (Fig. 2f). ETH-induced contractions are blocked by pre-treatment with epinastine, indicating that ETH-induced contractions occur via OA release.


**Additional file 2: Video S1.** Ovary contractions in response to OA. Dissected ovaries were treated with 50 μM OA (*blue arrow*); 10X speed. (MOV 99800 kb)
**Additional file 3: Video S2.** Ovary contractions in response to ETH. Dissected ovaries were treated with 10 μM ETH (*green arrow*); 10X speed. (MOV 96100 kb)


**Fig. 3:**
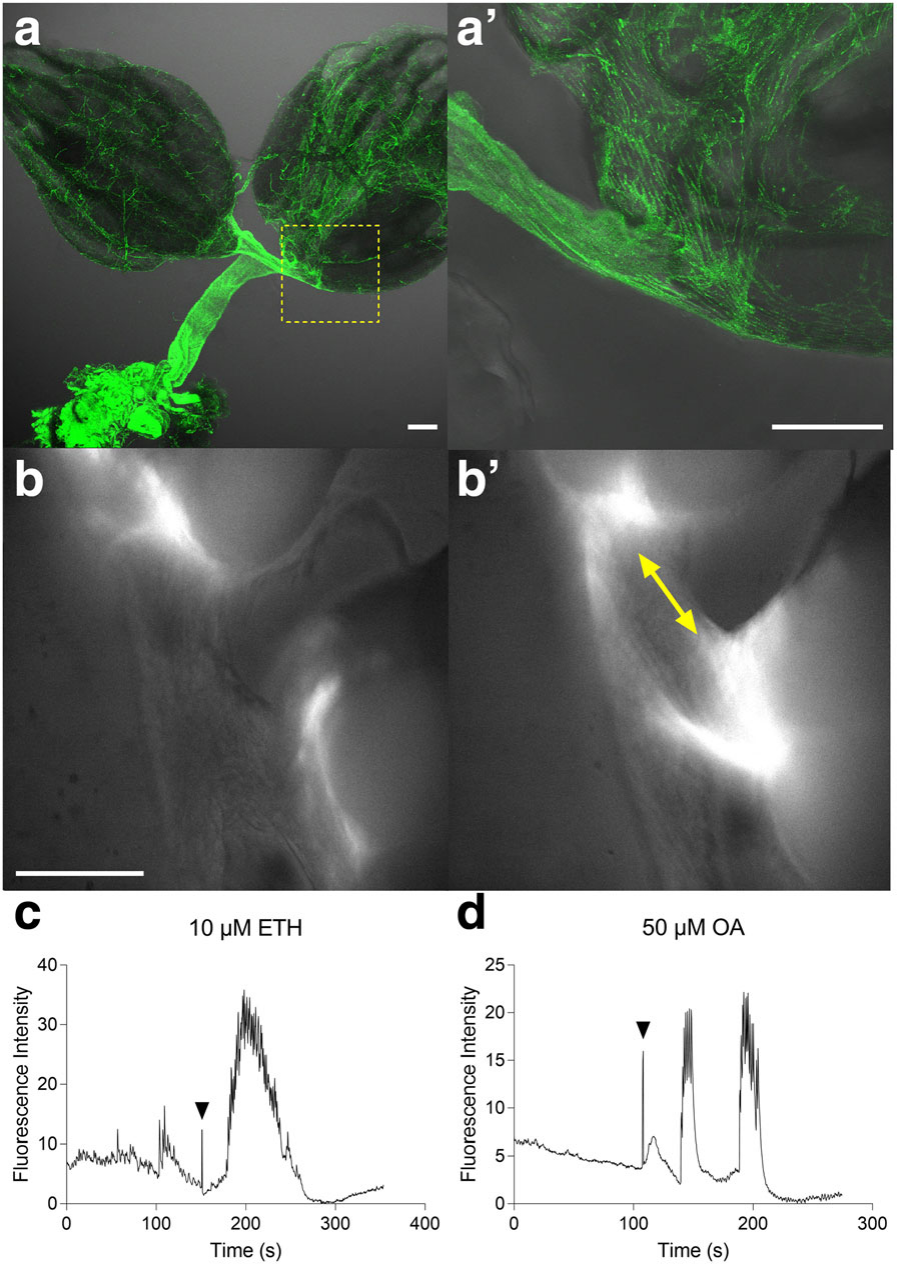
Basal ovary epithelium mobilizes calcium when contractions occur after ETH or OA treatment. **a** Expression pattern of *109-53-Gal4/UAS-mCD8-GFP* in the female reproductive tract (**a**) and at the base of the ovary (**a’**) (scale bars = 100 μm). **b** Calcium response in ovary epithelium (*109-53-Gal4/UAS-GCaMP6S*) before (**b**) and during (**b’**) contractions induced by ETH treatment (*yellow arrows* indicate calcium responses at the base of the ovary; scale bar = 100 μm). **c**, **d** Quantification of ovary fluorescence response to 10 μM ETH (**c**, *black arrowhead*) and 50 μM octopamine (**d**, *black arrowhead*) treatment


Expression of the calcium reporter GCaMP6S in ovary epithelia using the 109-53-Gal4 driver (Fig. 3a) allowed visualization of local calcium mobilization associated with ovarian contractions (Fig. 3b). Both ETH and OA treatments (Fig. 3c and d, respectively) evoke elevated calcium levels and bouts of strong contractions. Contractions occurring at the base of the ovary facilitate movement of stage 14 oocytes from the ovary into the oviduct (Additional file 4: Video S3 and Additional file 5: Video S4). In contrast, treatments with tyramine, glutamate, and proctolin all failed to stimulate ovary contractions (Additional file 1: Figure S1C–E).

**Additional file 4: Video S3.** Ovary contractions, calcium response, and ovulation after OA treatment. Dissected ovaries (*109-53-Gal4/UAS-GCaMP6S*) were treated with 50 μM OA (ovary base indicated by *red arrows*, treatment at *orange arrow*); 8X speed. (MOV 65500 kb)
**Additional file 5: Video S4.** Ovary contractions, calcium response, and ovulation after ETH treatment. Dissected ovaries (*109-53-Gal4/UAS-GCaMP6S*) were treated with 10 μM ETH (ovary base indicated by *red arrows*, treatment at *orange arrow*); 8X speed. (MOV 66800 kb)


**Fig. 4:**
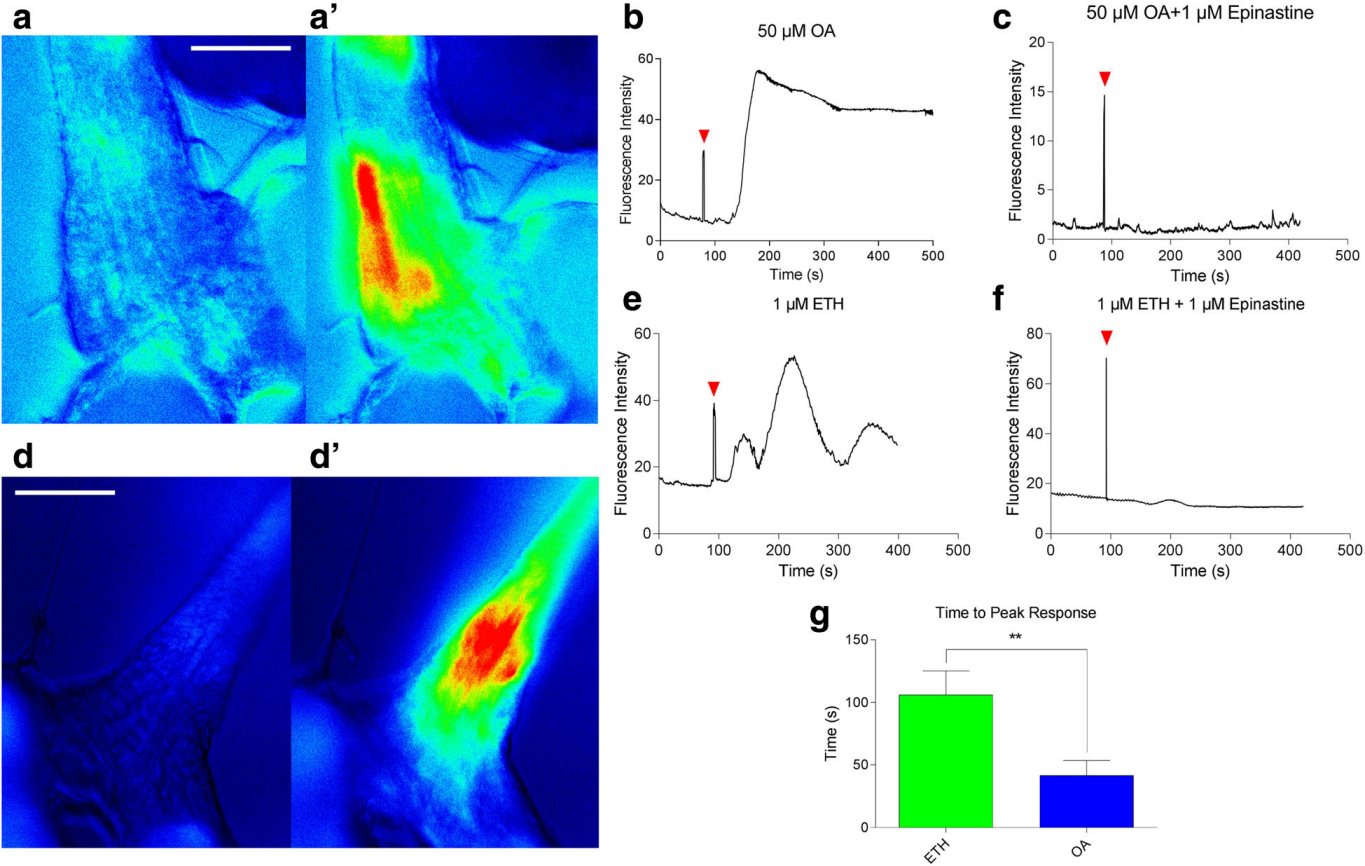
ETH mobilizes calcium in oviduct epithelium via octopamine release. **a**, **d** Oviducts of *OAMB-Gal4/UAS-GCaMP5* females before (**a**, **d**) and after 50 μM OA (**a’**) and 1 μM ETH (**d’**) treatment (scale bars = 100 μm). **b**, **e** Typical kinetics of the calcium responses to OA (**b**) and ETH (**e**). **c**, **f** Kinetics of oviduct calcium response to OA (**c**) and ETH (**f**) pre-incubated in 1 μM of the OA antagonist epinastine (treatment at *red arrowheads*). **g** Average latency to peak calcium response after ETH and OA treatment (*n* = 10) (biological replicates). Error bars represent SEM. **p < 0.01


OA is necessary and sufficient for relaxation of the oviduct, which is required for ovulation [22]. The relaxation response occurs via activation of two OA receptors (Octß2R and OAMB [21, 32]) in the oviduct epithelium, resulting in calcium mobilization, induction of nitric oxide synthase, and NO release [26]. Using the oviduct epithelium-specific OAMB-Gal4 driver, we examined oviduct calcium responses to ETH and OA exposure (Fig. 4a–f, Additional file 6: Video S5 and Additional file 7: Video S6). ETH and OA treatments stimulate strong, sustained calcium responses in oviduct epithelium localized to the juncture of lateral and common oviducts (Fig. 4a, d). In OA-treated females, the response is generally rapid and sustained for up to 20 min (Fig. 4b). On the other hand, ETH treatment elicits transient, oscillatory calcium responses with longer latency (Fig. 4g). Calcium responses to both ETH and OA are blocked by 1 μM epinastine (Fig. 4c, f). Time to peak calcium levels in response to ETH treatment are significantly longer than those for OA-induced responses (*p* < 0.05) (Fig. 4g). When pretreated with OA, oviduct calcium responses to additional ETH treatment are undetectable (Additional file 8: Figure S2A). As with ovary base contractions, tyramine, glutamate, and proctolin elicit no calcium mobilization response from oviduct epithelium (Additional file 8: Figure S2B–D).

**Additional file 6: Video S5.** Oviduct calcium response to OA treatment. Dissected oviduct (*OAMB-Gal4/UAS-GCaMP6S*) was treated with 50 μM OA (treatment at *orange arrow*); 8X speed. (MOV 56300 kb)
**Additional file 7: Video S6.** Oviduct calcium response to ETH treatment. Dissected oviduct (OAMB-*Gal4/UAS-GCaMP6S*) was treated with 10 μM ETH (treatment at *green arrow*); 8X speed. (MOV 96100 kb)


**Fig. 5:**
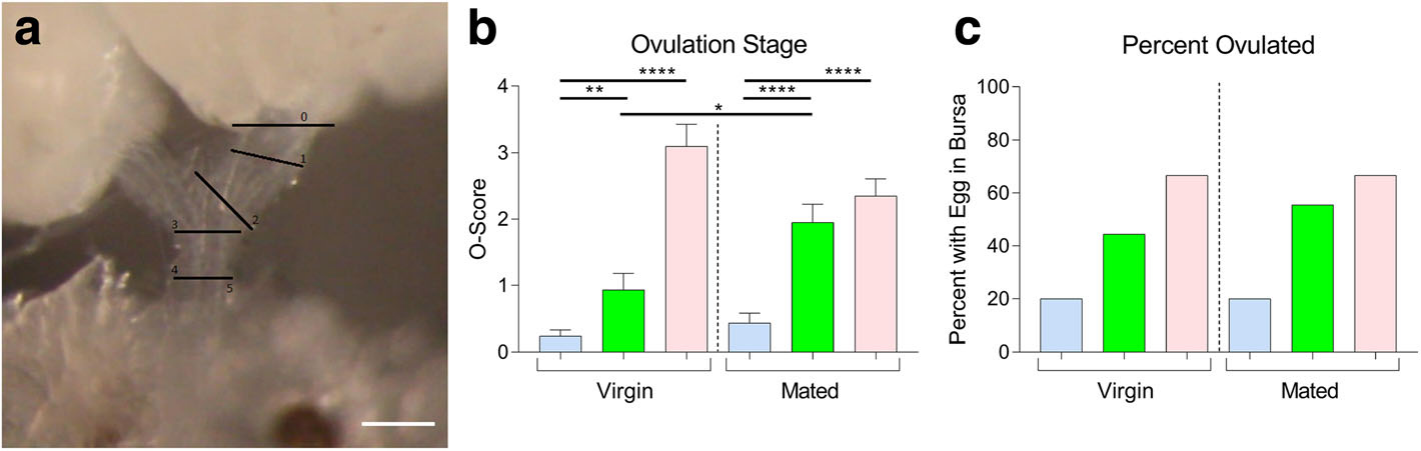
ETH activates ovulation in vivo*.* **a** Scoring system for in vivo ovulation. Ovaries were dissected, and oocyte position was scored according to which physiological plane was broken by the tip of the furthest oocyte (scale bar = 100 μm). **b** Quantification of oocyte distance in oviducts of females 90 min after injection with saline (*blue bars*), ETH (*green bars*), or OA (*pink bars*) (*n* = 20) (biological replicates). **c** Percent of the same females with an egg present in their bursa (percent ovulated) (*n* = 20) (biological replicates). Error bars represent SEM. *p < 0.05, **p < 0.01, ****p < 0.0001

We next examined whether injection of OA or ETH stimulates ovulation in vivo. Most in vivo studies of ovulation utilize a binary assay, whereby dissected ovaries are scored for the presence or absence of an oocyte in the oviduct or uterus [26, 33, 34]. In an attempt to measure ovulation rate, we scored the position of the ovulating oocyte in the oviduct (Fig. 5a) following injection of females with either saline, ETH, or OA. Injected individuals were flash-frozen 90 min post-injection. After freezing, the females were dissected and examined for the position of the ovulating oocyte in the reproductive tract. Both ETH and OA injection induce in vivo ovulation, measured either by oocyte position (Fig. 5b) or the presence of an oocyte in the bursa (percent ovulated; Fig. 5c). Mated females are more responsive than virgins to ETH but not to OA injection.

### ETH attenuates declining fecundity in heat-stressed females

**Fig. 6:**
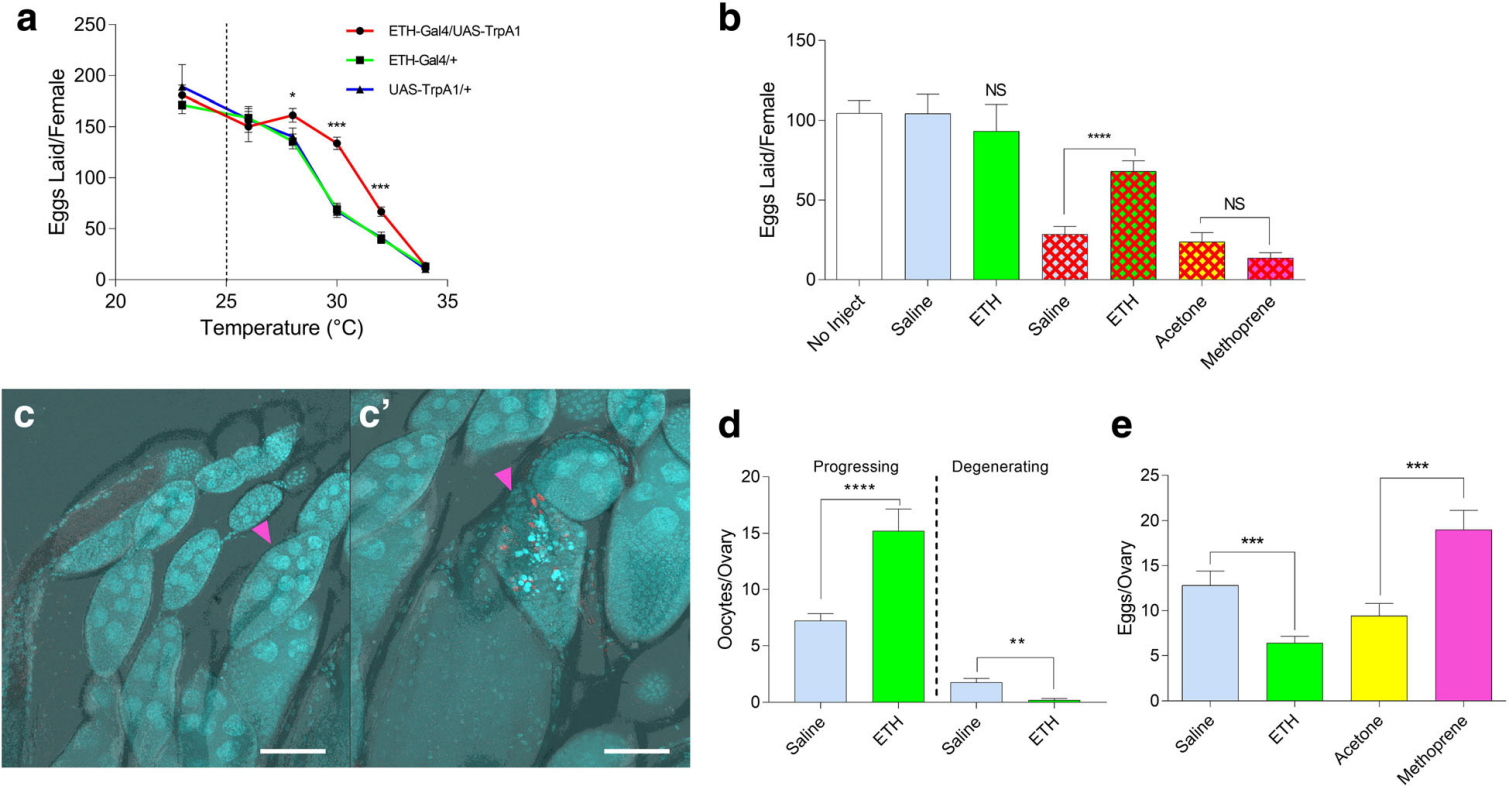
Heat stress-induced decreases in fecundity, oogenesis, and ovulation are mitigated by ETH injection. **a** Number of eggs laid by *ETH-Gal4/UAS-TRPA1* females exposed to various temperatures over a 3-day period compared to Gal4 and UAS controls (*n* = 20) (biological replicates). **b** Eggs laid by Canton-S faux heat-stressed females injected with ETH (*n* = 10) or saline (*n* = 10) at room temperature (*solid bars*) or mated females exposed for 1 h to 38 °C (*red cross hatch patterns*) and treated immediately thereafter with saline (*n* = 25), ETH (*n* = 25), acetone (*n* = 30), or methoprene dissolved in acetone (*n* = 35) (biological replicates). ETH treatment and heat stress had significant interaction with two-way ANOVA (*p* < 0.01). **c** Stage 9 progressing (*left, pink arrowhead*) and degenerating (*right, pink arrowhead*) oocytes from heat-stressed, ETH-injected (**c**) or saline-injected (**c’**) females, respectively. Preparations are stained terminal deoxynucleotidyl transferase dUTP nick end label (TUNEL, *red*) and DAPI (*blue*) (scale bars = 50 μm). **d** Quantification of progressing (stage 8/9 intact) and degenerating (stage 8/9 undergoing apoptosis) oocytes in saline and ETH (10 μM) injected females (*n* = 20) (biological replicates). **e** Number of mature eggs retained in ovaries of heat-stressed females injected with saline or ETH (10 μM) or treated topically with acetone alone or methoprene dissolved in acetone (*n* = 30). Error bars represent SEM. *NS*, *p* > 0.05; *p < 0.05, **p < 0.01, ***p < 0.001, ****p < 0.0001

Declining fecundity is a known consequence of elevated, thermally stressful temperatures [35]. Since impaired fecundity results from ETH deficiency [16], we asked whether ETH release from Inka cells under stressful conditions might mitigate the heat stress phenotype. We exposed females to increasing temperatures in the range 25 to 35 °C (Fig. 6a) and found that egg production in control flies declines significantly at 28, 30, 32, and 35 °C. At 28 °C, production drops from ~ 180 to 130, while at 34 °C production was less than 20 eggs. However, transient receptor potential channel (TrPA1)-mediated activation of Inka cells in *ETH-Gal4 > UAS-TrPA1* females elevated egg production significantly at 28, 30, and 32 °C.

We sought to confirm that ETH attenuates the egg production phenotype by assessing fecundity following injection of ETH1 into unstressed and heat-stressed mated females. When mated females are heat-stressed, fecundity declines for a number of days, with duration depending on the length of the heat stress [36]. We found that Canton-S females reduce egg production by ~ 80% following a 1-h, 38 °C heat stress (Fig. 6b). To avoid interfering with sperm storage, wild-type females were mated on day 4 and either heat stressed (1 h in a water bath heated to 38 °C) or faux stressed (1 h in water bath at room temperature) exactly 24 h later and then moved to room temperature for 3 days. Faux heat-stressed females injected with 20 pmol ETH showed no difference in fecundity compared to controls (Fig. 6b). When heat-stressed mated females were injected with 20 pmol ETH, we observed a significant attenuation of this decline (Fig. 6b), with females laying more eggs than the saline-injected controls. Two-way analysis of variance (ANOVA) confirmed an interaction between thermal treatment and injection of ETH (*p* < 0.01), arguing that ETH-induced stimulation of fecundity depends upon stress context.

These data suggest that ETH attenuates loss of fecundity under stressful conditions. One possible explanation for these observations is that heat stress causes a decline in circulating ETH. As oogenesis and fecundity depend upon juvenile hormone (JH), and ETH is an obligatory allatotropin [16, 37], we sought to determine whether treatment with the JH analog methoprene could attenuate heat shock-induced repression of fecundity. Methoprene treatment did not increase fecundity, suggesting that resumption of oogenesis without affecting ETH deficiency-induced anovulation cannot accelerate egg-laying under these circumstances (Fig. 6b).

### ETH attenuates heat stress-induced arrest of oogenesis and egg retention

ETH stimulates fecundity by stimulating progression of oogenesis via JH production [16]. In this study, we have shown that ETH activates octopaminergic neurons to facilitate ovulation. The ovarioles of heat-stressed mated females exhibit reduction of vitellogenic oocytes and increased rates of mid-oogenesis apoptosis, which is rare in unstressed flies [6, 16]. As expected, when we examined the mid-oogenetic oocytes (stages 8–10), we found that ETH injection into heat-stressed females increased the number of progressing oocytes while reducing the number going through apoptosis, with ETH-injected oogenesis comparable to that at unstressed levels (Fig. 6c, d, Additional file 9: Figure S3A, B).

Sugar starvation also is reported to promote mid-oogenesis apoptosis [13]. We found that ETH injection into sugar-starved flies rescues oogenesis (Additional file 9: Figure S3C, D). As mated females without an appropriate substrate on which to lay eggs rarely ovulate and oviposit [38], egg retention was not examined.

In the mated state, ovulation occurs soon after oogenesis is completed, and egg retention is minimal. However, upon exposure to heat stress, females retain a much greater number of stage 14 oocytes, but no clear mechanistic explanation has been reported [6]. We also observed that, while unstressed females retain very few eggs in their ovaries, heat stress increases the number of eggs retained threefold (Fig. 6e). ETH injection reverses egg retention (Fig. 6e), whereas treatment with methoprene increases the number of mature eggs retained, with increased egg production and sustained anovulation (Fig. 6e). Taken together, our data suggest that heat stress depresses fecundity by simultaneously arresting both oogenesis and ovulation.

### Heat and/or nutritional stress elevates ecdysone and lowers ETH

It is well established that heat stress leads to elevated ecdysone levels [6, 9]. High ecdysone levels during molts block ETH release by suppressing expression of the competence factor *βftz-f1* [17]. We have shown that heat-stressed females show hallmarks of ETH deficiency — arrested oogenesis and egg retention — and these phenotypes can be rescued by ETH treatment. We therefore asked whether elevated ecdysone under stressful conditions leads to ETH deficiency, which might explain the oogenesis and ovulation phenotypes described above.

**Fig. 7:**
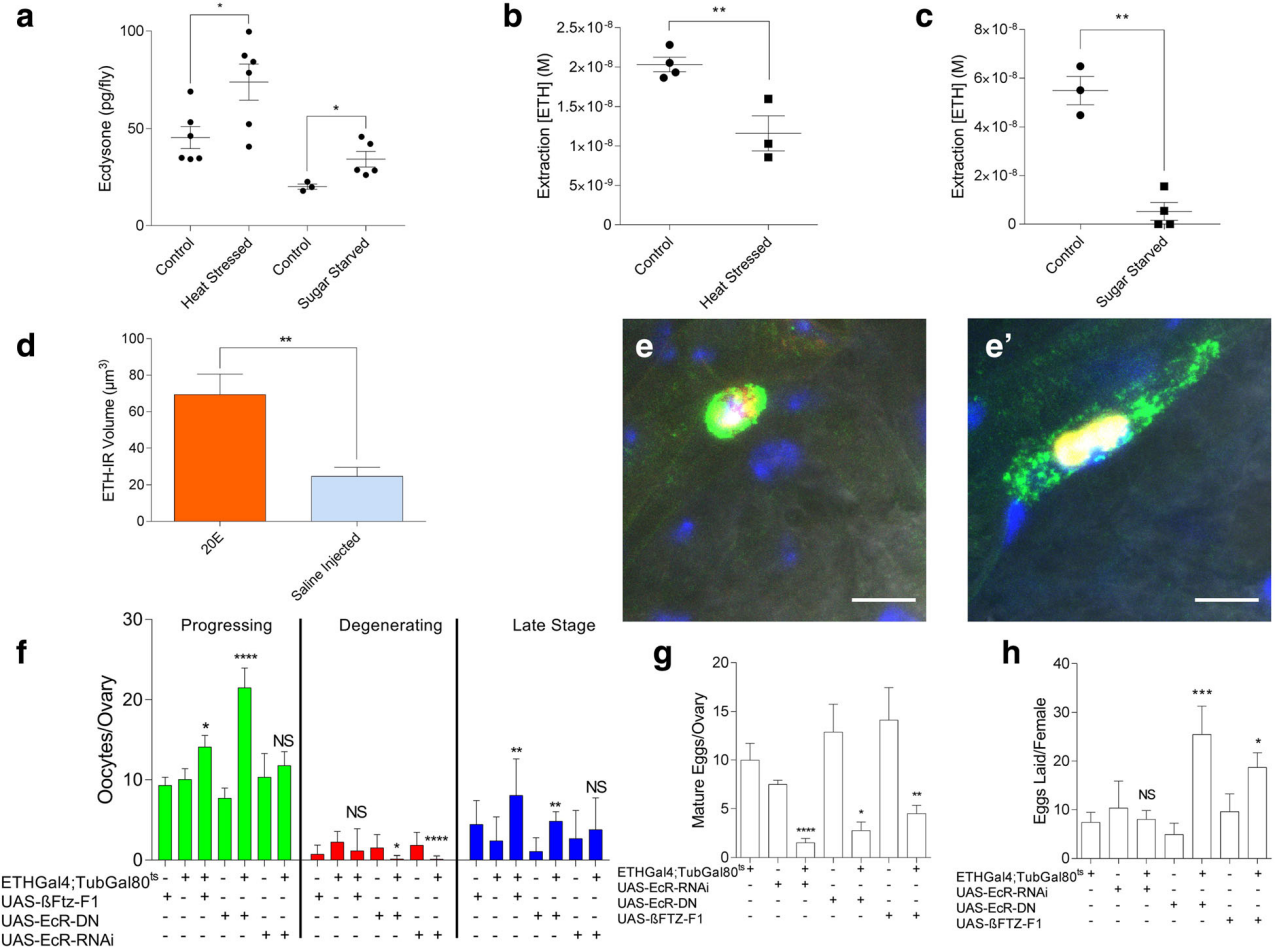
Ecdysone suppression of Inka cell secretory competence reduces ETH levels in hemolymph of stressed flies. **a** Ecdysone levels were determined by enzyme immunoassay (EIA) from unstressed/fed flies 24 h after heat stress or 24 h after starvation (*n* = 15 flies per point; each point represents a separate experiment). **b**, **c** ETH levels were determined by EIA from hemolymph extractions of unstressed mated females or 24 h after heat stress (**b**, see Additional file 10: Figure S4E) and after 24 h of sugar starvation (**c**, see Additional file 10: Figure S4F) (*n* = 50 flies per point; each point represents a separate experiment). **d** Volume of ETH immunoreactivity in A4 Inka cells from saline- or 20-hydroxyecdysone (*20E*)-injected females (*n* = 20). **e** ETH-like immunoreactivity of Inka cells (*green*) observed in *ETH-Gal4/UAS-Red Stinger* females 24 h after injection of either saline (**e**) or 36 ng of 20E (**e’**) (scale bars = 10 μm). **f**–**h** Heat-stressed females of genotypes *ETH-Gal4;Tubulin-Gal80*^*ts*^*/UAS-ßFTZ-F1*, *ETH-Gal4;Tubulin-Gal80*^*ts*^*/UAS-EcR-DN*, *ETH-Gal4;Tubulin-Gal80*^*ts*^*/UAS-EcR-RNAi*, and genetic controls scored for oogenesis (**f**) stages 8–9 progressing (*green*), degenerating (*red*), and late stage (10–13) oocytes (*blue*), (**g**) egg retention, and (**h**) eggs laid during the 3 days after heat stress (*n* = 30) (biological replicates). Error bars represent SEM. *NS*, *p* > 0.05; *p < 0.05, **p < 0.01, ***p < 0.001, ****p < 0.0001

We found that heat stress or sugar starvation indeed elevates ecdysone levels (Fig. 7a). We examined whether elevation of ecdysone under conditions of heat stress affects circulating ETH levels by performing an enzyme immunoassay (EIA). To avoid rupturing Inka cells, we extracted the hemolymph by making incisions in the ventral abdomen and dorsal thorax and bled the animals according to procedures described in the Methods section. The hemolymph extraction protocol was validated by injecting known quantities of ETH into mated females. We found that the quantity of ETH extracted increased in proportion to the dose injected (Additional file 10: Figure S4A). This procedure also allowed us to estimate the amount of hemolymph-borne ETH recovered (2.111 ± 0.221%) and relative levels of circulating ETH in mated females (950 nM at time of extraction).

Circulating levels of ETH-like immunoreactivity are significantly reduced under heat stress conditions (Fig. 7b). Likewise, sugar-starved females show a significant drop in ETH levels (Fig. 7c). In contrast, wet starvation resulted in a decline of ecdysone levels (Additional file 10: Figure S4B) but an elevation of ETH (Additional file 10: Figure S4C). Taken together, these data suggest an inverse relationship between levels of circulating ecdysone and ETH.

### Ecdysone repression of ETH release is necessary and sufficient for heat stress-induced attenuation of oogenesis and ovulation

Elevated ecdysone levels are associated with activation of apoptosis during mid-oogenesis, leading to arrest of vitellogenesis, progression of post-stage 7 oocytes to latter stages of oocyte development, and decreased fecundity [12, 39–41]. Recent evidence suggests that high ecdysone levels also may inhibit the follicle rupture necessary for ovulation [12, 14], but the mechanistic explanation for these phenomena remains lacking.

High ecdysone levels associated with molting induce ETH production but block its release, causing Inka cells to increase in size and ETH-like immunoreactivity [17, 42]. To determine whether ecdysone has similar effects on Inka cells during the adult stage, we injected mated females with the biologically active form of ecdysone, 20-hydroxyecdysone (20E) (20 pM) and found that Inka cells increased in volume and ETH-like immunoreactivity (Fig. 7d, e).

These results suggest that high levels of ecdysone are associated with increased synthesis [16] but suppressed release of ETH during adulthood. Prior to ecdysis, high concentrations of ecdysone suppress expression of *βftz-f1*, which is required for secretory competence of Inka cells [17]. We therefore examined whether we could rescue heat-stress-induced arrests in oogenesis, ovulation, and egg-laying by disrupting ecdysone-mediated repression of Inka cell secretory competence. Using Inka cell-specific GAL4 drivers for (1) EcR knockdown, (2) overexpression of an EcR dominant negative isoform, and (3) overexpression of *βFTZ-F1*, females were exposed to heat stress and tested for egg production and physiological markers of ETH deficiency. Test females showed a clear increase in progression beyond mid-oogenesis (Additional file 10: Figure S4E, F) and fewer mature eggs retained in their ovaries compared to controls (Fig. 7g). Ovariole profiles thus suggest that egg development and ovulation are rescued by these manipulations. Furthermore, expression of either dominant negative EcR or *βftz-f1* led to significant increases in egg production under heat stress conditions (Fig. 7h). These findings confirm that stress-induced elevation of ecdysone levels reduces secretory competence of Inka cells, leading to ETH deficiency.

## Discussion

Evidence presented here establishes a new paradigm for *Drosophila* reproduction, wherein stressful conditions arrest egg production via a hormonal cascade involving reciprocal ecdysone and ETH signaling. As steroid levels fluctuate in response to stress, so too does ETH, a consequence of steroid-regulated changes in Inka cell secretory competence. ETH activates two downstream targets: the JH-producing corpus allatum and modulatory OA neurons innervating the ovary and oviducts. We characterize the nature of ETH dependence, and assign function and context to a newly recognized hormonal axis governing reproductive responses to stress.

Our previous report showed that ETH is an obligatory allatotropin, promoting oogenesis and fecundity through JH production; consequently, ETH deficiency results in low JH levels and arrested oogenesis [16]. In the present work we demonstrate for the first time that ovulation of stage 14 oocytes depends upon ETH activation of OA neurons innervating the ovary and oviduct. We also offer a comprehensive explanation for the change in distribution of vitellogenic oocytes reported in EcR mutants or under conditions of high or low ecdysone, depending on stress levels [6, 12, 43, 44]. We show that ETH deficiency or ETHR knockdown results in accumulation of stage 14 oocytes in the ovary due to ovulation block [21, 22, 26] and provide for the first time a mechanistic link between altered endocrine state and ovulation.

### ETH regulates OA signaling in the female reproductive tract

We have demonstrated that ETH promotes ovulation through activation OA neurons to induce contractions in the ovary and relaxation of the oviducts. It is interesting that ETH triggers calcium dynamics in vitro on distal axonal projections, suggesting ETH-stimulated OA release results from direct action of ETH on axons and/or nerve terminals. While ovary contractions in response to ETH exposure occur in both virgin and mated females, we chose virgin females for analysis due to higher spontaneous contractile activity in mated females. This is likely due to actions of ovulin after insemination, which stimulate outgrowth of octopaminergic neurons innervating the oviduct [27]. In virgin females, concentration-dependent ETH actions on the ovary (low micromolar) are in the range predicted for activation of ETHR-A receptors [31].

Acting through OA neurons, ETH mobilizes calcium in the epithelium enveloping the ovary, initiating bursts of contractions in the peritoneal sheath at the base of the ovary associated with ovulation. Although bath-applied ETH and OA are both sufficient to induce calcium mobilization in the oviduct epithelium, they induce distinctive response patterns. OA causes a rapid, sustained calcium wave with a slowly waning plateau following the peak response. ETH actions occur with longer latency and induce oscillatory calcium dynamics, which could be a consequence of periodic synaptic reuptake of OA by nerve terminals. No changes in intensity were observed between treatments or at different doses, suggesting a possible threshold effect. It is also interesting to note that calcium waves spread through the epithelial layer (Additional file 7: Video S6), suggesting that the epithelium is a functional syncytium, which undoubtedly aids in coordination of relaxation.

Injection of mated females with either ETH or OA induces ovulation in vivo, whereas injected virgin females respond much more weakly. In order for ovulation to occur, OA causes follicle rupture inside the ovaries, a process requiring one to several hours ex vivo [14, 45]. We hypothesize that mated females are in the proper endocrine state for ovulation, and thus follicle rupture may already be in progress before application of ETH or OA. As follicle rupture is the critical first step for egg-laying, this limiting factor would explain the length of time (up to 60 min) elapsed after physiological levels of ETH/OA are reached for in vivo ovulation to occur, given that ovary contraction and oviduct relaxation occur within seconds.

We also examined agents previously implicated in oviduct contractions, including tyramine, glutamate, and proctolin. While the ineffectiveness of tyramine and glutamate is not surprising, the negative result with proctolin is at variance with prior literature [19]. Examination of proctolin-induced contractions revealed that they are localized to the distal tip (germaria) of the ovaries. Moreover, proctolin does not stimulate ovulation in vitro. It appears that the role of proctolin in *Drosophila* ovaries is more limited than in the well-studied locust oviduct [46].

### Stress (heat and yeast deprivation) elevates ecdysone, creating circulating ETH deficiency

We have shown that elevated ecdysone levels in response to heat and nutritional stress are associated with a drop in circulating ETH levels. We previously hypothesized that the Inka cell secretory competence model governing ecdysis signaling during developmental stages may persist into adulthood [16]. The results presented here support this hypothesis.

Both stress and ETH deficiency have similar consequences for reproduction, namely arrested oogenesis and reduced ovulation, resulting in increased stage 14 egg retention and lower egg production. Progression of mid-oogenetic oocytes is directly correlated with JH levels [12, 18, 47], while OA release from reproductive tract neurons is necessary for ovulation [21, 25]. Here we show that arrested oogenesis and ovulation contributing to the ovariole profile observed in heat-stressed flies [6] can be explained by ETH deficiency, which has a dual role in regulating JH levels and activity of OA neurons innervating ovaries and oviducts. Indeed, arrest of both oogenesis and ovulation deficiencies can be rescued by ETH, either through TRPA1 activation of Inka cells or direct injection of ETH1.

We examined the mechanism through which elevated ecdysone leads to ETH deficiency by performing rescue experiments designed to (1) suppress steroid signaling in Inka cells and (2) express the transcription factor *ßFTZ-F1*, which confers secretory competence of Inka cells and is suppressed by high ecdysone levels [17]. Although somewhat variable in their effectiveness, these manipulations resulted in clear rescue of oogenesis and ovulation in heat-stressed females, confirming that the thermal stress response operates through the influence of ecdysone on Inka cell secretion.

### Implications of the ecdysone/JH balance on oogenesis and ovulation

**Fig. 8:**
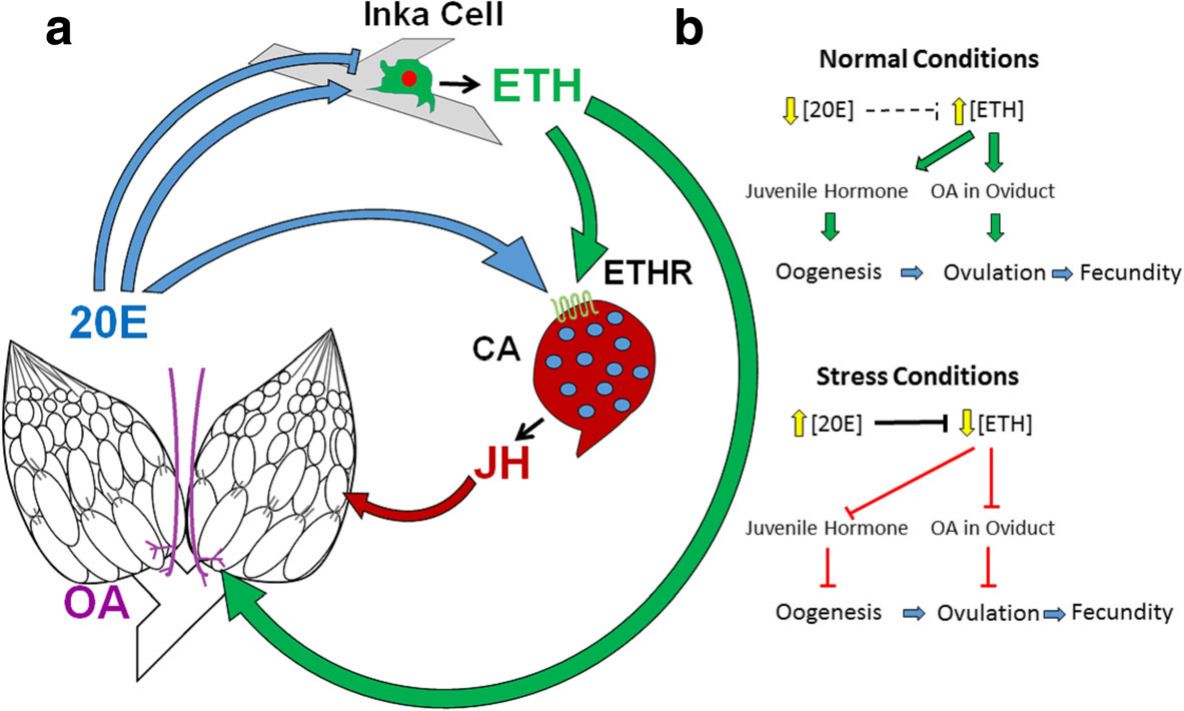
Model depicting *Drosophila melanogaster* adult endocrine axis. **a** Direct influences of 20E, ETH, JH, and OA on one another and on the female reproductive system. **b** Model depicting how circulating ecdysone can regulate fecundity via ETH and its targets under normal and stress conditions. Fecundity depends hierarchically on oogenesis and ovulation

We found that methoprene treatment increases progression of oogenesis but does not increase oviposition in stressed animals. These results are similar to those reported previously [12]. In fact, we observed a significant increase in eggs retained after methoprene treatment, suggesting that synthesis of mature eggs resumes with JH treatment, but ovulation remains impaired under conditions of elevated ecdysone and ETH deficiency. This suggests that ovulation provides a gating mechanism under stressful conditions, limiting egg production while conditions are suboptimal (Fig. 8). A recent report suggested that normal ecdysone levels stimulate follicle rupture and ovulation, but that elevated levels inhibit follicle rupture [14]. The present work provides an additional mechanism for suppression of ovulation associated with elevated ecdysone levels: repression of ETH release leading to reduced OA neuron activity.

Drosophila responds to courtship, nutritional, chemical, and thermal stress with elevation of ecdysone levels [9, 10, 48, 49]. Here we show that sugar starvation and heat stress depress ETH levels in the hemolymph. We only observed a relatively small reduction in ETH levels, but this could be due to two reasons. Our extraction method required dilution of the hemolymph in order to minimize coagulation. This may pare any differences between samples in ETH immunoreactivity depending on the success of the extraction. Second, we were operating at the limit of quantification (LOQ) for the ETH1 antiserum. Consequently, concentrations under 18 nM could not be accurately quantified, and thus heat-stressed females may have even lower ETH than the EIA could indicate.

It is interesting to note that wet starvation reduces ecdysone levels and increases ETH levels, whereas sugar starvation increases ecdysone levels [39] and, as we show here, increases ETH levels. Wet-starved females were precisely synchronized in mating on day 4, and began starvation (no nutrient source, wet KimWipe) 24 h later for an additional 24 h. In order to mimic conditions described by Terashima et al. [39], amino acid-deprived females were group-raised until day 3, and groups were placed on agar + 10% sucrose for 24 h. Mating was not controlled in sugar-starved females, though it is known to influence ecdysone levels dramatically in the short term [50]. This, in addition to our slightly different age (6 days after eclosion at time of measurement vs 4 days) and different method of starvation (wet starvation vs agar medium and 5% sucrose), may also change endocrine state. While these discrepancies may all be contributing factors, arguably the most interesting result is that ecdysone decrease led to elevated circulating ETH. This adds credence to the hypothesis that ETH and ecdysone levels are generally inversely correlated.

Unique stresses may garner different endocrine responses because different types of cues require differential behavioral adaptation. The ability of a hormone to coordinate a wide variety of target tissues to change in state makes it a perfect tool for stress adaptation. As an organism encounters a new type of stress, they may adapt a new endocrine state to coordinate a tissue-wide response. Many hormones in closely related insects play markedly different roles, which evolve as rapidly as behavioral niches, but an endocrine core in E-ETH-JH is highly conserved, similar to the hypothalamic-pituitary-gonadal (HPG) axis among vertebrates. A hormonal network with competence to adjust reproductive output in response to environmental changes is undoubtedly a common phenomenon among multicellular organisms. The discovery of a stress response hormonal axis and, more aptly, a peptide hormone with the potential to alleviate stress-induced deficits in reproduction could be of particular relevance to the honey bee *Apis mellifera.* In recent years, *Apis* reproductives have been producing fewer progeny due to a variety of stressors, including temperature extrema [51, 52]. While proctolin has already been found to be a short-term reproductive stimulant in *Apis* queens [53], ETH is attractive as it can alter JH levels, which in turn may rescue poor pheromone production, the proximal cause of supersedure [54–56].

## Conclusions

In summary, we have demonstrated that the peptide hormone ETH is critical for proper functioning of octopaminergic neurons innervating the reproductive tract and, consequently, ovulation. Inka cells, the sole source of ETH, appear to be tightly regulated by hemolymph ecdysone levels. Stress-induced elevation of ecdysone levels leads to ETH deficiency, resulting in reduced reproductive output through arrest of oogenesis and ovulation. This provides the first mechanistic explanation connecting known endocrine perturbation and anovulation and sets the context for further examination of endocrine regulation of stress and reproduction in insects.

## Methods

### Flies

Flies used for immunohistochemistry, calcium imaging, and corpora allata ETHR silencing were raised at 23 °C on standard cornmeal-agar media under a 12:12 h light:dark regimen. Inka cell-ablated and EcR-interrupted flies were raised as described previously [16]. Inka cell-blocked and conditional ETHR-silenced or EcR-dominant-negative flies (genotype *ETH-Gal4;tubulin-Gal80*^*ts*^) were raised at the Gal80^ts^ permissive temperature (18 °C) and moved to the nonpermissive temperature (28 °C) after eclosion until day 4. The use of double-stranded RNA constructs for silencing of ETHR (UAS-ETHR-Sym; UAS-ETHR-IR2 line (VDRC transformant ID dna697)) was described recently [57]. *Bwk*^*tqs*^*-Gal4* flies were obtained from Dr. Enrique Reynaud (Instituto de Biotecnología, Mexico City, Mexico). *OAMB-Gal4* was obtained from Dr. Kyung-An Han (University of Texas, El Paso, TX, USA). *UAS-ßFTZ-F1* was described previously [17]. *ETHR-Gal4* was obtained from Dr. Ben White (National Institute of Mental Health, Silver Spring, MD, USA). All other fly lines were obtained from the Bloomington Stock Center (Indiana University, Bloomington, IN, USA): *Tdc2-Gal4* (BS no. 9313), *UAS-TrpA1* (BS no. 26264), *UAS-Red Stinger* (BS no. 8574), *UAS-mCD8-GFP* (BS no. 5137), *UAS-rpr* (BS no. 8524), *UAS-GCaMP6S* (BS no. 42746), *UAS-GCaMP5* (BS no. 42037), *TubP-Gal80*^*ts*^ (BS no. 7017), *ETH-Gal4* (BS no. 51982), *UAS-EcR-RNAi* (BS no. 37059), *UAS-EcR.B1* (BS no. 6869).

### Immunohistochemistry

#### Labeling of ETHR-expressing octopaminergic neurons

Ovaries were removed from group-raised females of the genotype *Trojan ETHR-Gal4/UAS-mCD8-GFP* and immediately placed in 4% paraformaldehyde for 55 min. Once fixation was complete, ovaries were rinsed briefly in phosphate-buffered saline (PBS) with 5% Triton-X (PTX) and five times for 10 min in 0.5% PTX. After washing, ovaries were blocked for 5 h in 0.5% PTX + 5% normal goat serum (NGS) and then swapped for 0.5% PBST + 5% NGS containing mouse anti-GFP primary (Invitrogen, 1:500) and rabbit anti-tyrosine decarboxylase 2 (1:200, Covalab, Atlanta, GA, USA). After two overnights, samples were washed five times and Alexa Fluor 488-labeled goat anti-mouse IgG (Invitrogen), Alexa Fluor 555 goat anti-rabbit (Invitrogen), and 0.5 mg/ml 4’,6-diamidino-2-phenylindole (DAPI) in 0.5% PBS-Tween20 (PBST) + 5% NGS were added. After 4 days, samples were washed five times, mounted in Aqua Poly/Mount (Polysciences Inc., Warrington, PA, USA), and imaged on a Leica SP5 confocal microscope.

#### Inka cell staining

Flies were mated on day 4, heat-stressed or injected on day 5, and opened and fixed 24 h later. Immunohistochemistry for visualization of the Inka cells was performed as described previously [16]. After staining, we constructed image stacks using 1-μm sections. The volume of Inka cells was measured using the Volume Viewer and region of interest (ROI) tools in ImageJ. The ETH-IR volume is the total volume minus the volume of the DAPI and Red-Stinger-labeled nucleus.

### Treatments

#### Heat stress

Flies of indicated genotypes were mated on day 4 and 24 h later were aspirated into an empty glass culture tube (no more than five females per tube) and submerged in a 38 °C hot water bath for 1 h. Flies were either injected first or placed on a food plate for egg-laying immediately afterward.

#### Starvation

Wet starved females were mated on day 4. After 24 h females were placed in a vial with a wet KimWipe for 24 h before extraction. Sugar-starved females were group-raised until day 3 and placed on 3% agar + 10% sucrose plates for 24 h and extracted. For injections, sugar-starved females were injected abdominally with either ETH or fly saline alone on day 3 and placed on 3% agar + 10% sucrose plates for 48 h before assessment of fecundity and oogenesis.

#### Injections/topical application of methoprene

Injections or topical application of methoprene/acetone was performed immediately after heat shock. Females were anesthetized on ice and placed on an Echotherm chilling plate. For methoprene treatment, females were treated as described previously [16] before being given 72 h for egg-laying. For injections, females were anesthetized and injected with 50 nl of 100 μM ETH in the abdomen.

### Egg retention and egg-laying

Female flies of the indicated genotype were isolated in 1-cm culture tubes with standard fly food at eclosion. For egg-laying, after 4 days flies were mated with wild-type males and re-isolated for 24 h to store sperm. After 24 h, females were heat shocked and injected before being moved to apple juice-agar-yeast plates for 3 days to lay eggs as previously described (or see [16]). TRPA1 flies were raised at 23 °C for 4 days and moved to an incubator of indicated temperature on an apple juice plate for 3 days. For egg retention, virgin females were dissected at 3:00 pm, ovaries were opened up, and mature, stage 14 oocytes in the ovaries were counted and compared.

### Calcium imaging

#### Octopaminergic neurons

Group-raised females of the genotype *Tdc2-Gal4/UAS-GCaMP6S* > *UAS-mCD8-RFP* were reared at 28 °C after eclosion until day 5. At day 5, females were cold-anesthetized and ovaries were removed in fly saline and immediately added to 90 μl of fly saline + 10 μM epinastine and 50 μM Mn^2+^ to prevent contraction-associated movement of the sample. The sample was put on a glass cover slip and onto an SP5 inverted confocal microscope, recorded at 40X. After 100 s of habituation, ovaries were treated with 10 μl of 100 μM ETH1 (final concentration 10 μM) and recorded for 10 min.

#### Epithelial response

Group-raised females of the genotype *OAMB-Gal4/UAS-GCaMP6S* (oviduct epithelium) or *109-53-Gal4 > UAS-GCaMP6S* (ovary epithelium) were reared at 28 °C after eclosion until day 5. We used an imaging setup consisting of a Polychrome V monochromator (TILL Photonics/FEI) as light source and a TILL Imago charge-coupled device (CCD) camera. The microscope (Olympus Model BX51WI) was equipped with a 40X W NA 0.8 objective. Binning on the chip (8 × 8) was set to give a spatial sampling rate of 1 μm/pixel (image size 172 × 130 pixels, corresponding to 172 and 130 μm). Images were taken at a rate of 4 Hz. The excitation wavelength was 488 nm, and the exposure time was 25 ms. Fluorescent light passing an excitation filter (370–510 nm) was directed onto a 500-nm DCLP mirror followed by a 515 LP emission filter for enhanced green fluorescent protein. Timing of treatment was performed as above; the bath was 180 μl and 20 μl for treatment as indicated.

### Ovary contractions

For dose-response curves, ovaries were removed from cold-anesthetized, Canton-S virgin female flies and immediately added to 180 μl of fly saline, then placed under a stereomicroscope with a Canon EOS Rebel T5i video camera. We added 20 μl of 10X the indicated dose of ETH or OA to the bath at 2 min after recording began, and recording continued until 4 min. Videos were reviewed single blind, and the number of contractions during the 2 min prior to and after treatment was tallied. For OA neuron ETHR silencing, videos were prepared in the same fashion, but contractions for each 30-s interval of the video were tallied, averaged, and graphed over time to analyze contractions over time. Epinastine (200 μM) was added to the saline just before the ovaries when indicated.

### Ovulation

In vivo ovulation was assessed by cold-anesthetizing and injecting group-raised day 4 females abdominally with 36 nl of the indicated amount of ETH or OA dissolved in fly saline or saline alone. After 90 min of recovery time, flies were flash-frozen with dry ice, and the cuticle was removed from around the ovaries to view the position of the egg. For each fly, the furthest ovary along the oviduct was scored for position and recorded.

### Oogenesis

After 3 days of egg-laying, ovaries were removed from flies of various treatments and DAPI/terminal deoxynucleotidyl transferase dUTP nick end label (TUNEL) stained using the previously described protocol [16].

### Enzyme immunoassay (EIA)

#### ETH extraction

Canton-S females were mated on day 4, and 24 h later were subjected to heat stress, wet or sugar starvation, or injection with ecdysone. After an additional 24 h, groups of 8–12 females were cold-anesthetized on ice and placed in wells of a round-bottom glass slide on a chilling plate (Echotherm, Torrey Pines Scientific, San Diego, CA, USA) set to 2 °C. Wings and tarsi were removed, and flies were moved to a dry well. To leach out the polar ETH from the hemolymph and prevent coagulation, 1 μl of molecular grade water per fly was added to the bottom of the well, and flies were opened on the dorsal thorax and ventral abdomen, taking care not to rupture tissue, including trachea. Flies were each gently depressed once to ensure mixing of hemolymph and water, removed, and placed head down (to avoid genital tract contamination) in a 1-ml centrifuge tube, which was previously perforated several times at the bottom with a 27.5 gauge needle. The remaining hemolymph-water mixture was removed and added to the tube, and the tube was centrifuged for 5 min at 5000 g. The flow-through was collected and immediately moved to storage at –80 °C. Once 50 flies were collected, the tube was spun at 16,000 g, and the supernatant was removed for analysis by EIA.

#### Ecdysone extraction

Females were prepared as above, except that each experiment pooled 15 flies. Extraction differed in that free ecdysone was separated from its glandular source by removal of the ovaries and decapitation. The remaining body parts were homogenized in 300 μl of methanol and centrifuged at 16,000 g, and the supernatant was removed and spun for 3 h in a vacuum centrifuge. The residue was re-suspended in EIA buffer.

#### Enzyme immunoassay process

Antiserum to ETH1 was raised in rabbits against the N-terminal sequence of the peptide by conjugating the antigenic amino acid sequence (Asp-Asp-Ser-Ser-Pro-Gly-Phe-Phe-Leu-Lys-Iso-Thr-Lys-Cys) to bovine serum albumin (BSA). The enzyme immunoassay was performed as described previously [58]. For ETH determinations, 25 μl of extract was added 1:1 to 2X EIA buffer. Anti-ETH1 antibody was pre-incubated with the cysteine-BSA conjugate used at 1:10,000 concentration; horseradish peroxidase-ETH conjugate was used at 1:500 for establishment of a standard curve and ETH determination. For ecdysone determinations, groups of 15 ovary-less females were homogenized in methanol and centrifuged at 16,000 g. The supernatant was removed and vacuum evaporated in a Speed-Vac vacuum centrifuge. The homogenate was re-suspended in EIA buffer with BSA. Anti-ecdysone antibody [59, 60] and HRP conjugate were used at 1:100,000 and 1:10,000, respectively.

#### Analysis

The 96-well plate design included increasing concentrations of known concentrations of ETH or 20-E in EIA buffer + NGS or BSA, respectively, in triplicate. 3’,3’,5’,5’-tetramethylbenzidine (TMB) peroxidase substrate + peroxidase (KPL) was added to each well and allowed to react for ~ 12 min until the reaction was terminated with 1 M phosphoric acid. The absorbance at 450 nm was immediately read, and the absorbance of standards was plotted and interpolated using a four-parameter logistic equation, used to predict concentrations of unknowns.

### Statistical analysis

All sample groups were tested for normal distribution using quantile-quantile plotting (R) and the Shapiro-Wilk test (Prism). For experiments involving Gal4/UAS-driven expression of a transgene, test females were compared to genetic controls with one-way ANOVA. As all samples were normally distributed, comparison between groups with different chemical treatment or a given group before and after treatment was performed with a Student’s *t* test. Egg-laying data for heat-stressed and ETH-injected females were organized into a 2 × 2 matrix, and the interaction between heat stress and treatment was analyzed with a two-way ANOVA comparison.

## Additional files

Additional file 1: Figure S1. Tyramine, proctolin, and glutamate do not stimulate contractions at the base of the ovary. (A, B) Response to saline in octopaminergic neurons of the (A) oviduct (*blue arrowhead*) and ovary (*orange arrowhead*) (scale bar = 100 μm). (B) Nerve terminal fluorescence intensity from (A) over time, before and after treatment. (C–E) Fluorescence intensity in basal ovary epithelium (*109-53-Gal4/UAS-GCaMP6S*) before and after tyramine (C), glutamate (D), and proctolin (E) at indicated doses (treatment at *red arrowheads*). (JPG 695 kb)

Additional file 8: Figure S2. OA, but not tyramine glutamate or proctolin, causes calcium release in the oviduct epithelium. (A) Fluorescence intensity of oviduct epithelium (*OAMB-Gal4/UAS-GCaMP6S*) after saturating dose of octopamine (500 μM, *red arrowhead*) and subsequent ETH treatment (10 μM, *green arrowhead*). (B–D) Oviduct epithelium response to treatment (*red arrowheads*) with 10 μM tyramine (B), 20 μM glutamate (C), and 10 μM proctolin (D). (JPG 418 kb)

Additional file 9: Figure S3. ETH injection rescues oogenesis in heat-stressed females and oogenesis and fecundity in sugar-starved, mated females. (A, B) DAPI-stained ovary from heat-stressed, saline-injected female (A) and one injected with ETH (B), mid-oogenetic oocytes, *yellow arrowheads* (scale bar = 500 μm). (C) Egg-laying of Canton-S females during 24 h of sugar starvation after injection with saline (*n* = 23) or ETH (*n* = 19) just prior. (D) Staging of mid-oogenesis oocytes dissected from ETH- or saline-injected females after 24 h of starvation (*n* = 20) (biological replicates). Error bars represent SEM. *p < 0.05, **p < 0.01. (JPG 449 kb)

Additional file 10: Figure S4. Enzyme immunoassay (EIA) validation, hormonal state change with wet starvation, and genetic rescue of heat stress-induced oogenesis arrest. (A) EIA after injection of saline, 10 ng or 50 ng ETH, along with predicted concentrations based on relative quantity injected (*red arrows*). Recovery was found to be ~ 2% of the predicted ETH concentration in the hemolymph, assuming a total blood volume of 1 μl. (B, C) Hemolymph ecdysone (B) and ETH (C, ETH determination Curve 1) levels in unstressed and wet starved females. (D–F) EIA standard curves used for quantification of ecdysone (D) and ETH (E, F) levels from Fig. 6a–c, respectively (*r* square > 0.99). (G, H) Example ovaries from heat-stressed females of the genotype *UAS-ßFTZ-F1* (G) and *ETH-Gal4;TubulinGal80*^*ts*^*/UAS-ßFTZ-F1* (H). *Red arrowheads* indicate mature eggs retained, while *yellow arrowheads* indicate vitellogenic, progressing oocytes (scale bars = 500 μm). (JPG 1.14 mb)

## Abbreviations

20E: 20-Hydroxyecdysone
CA: Corpora Allata
EIA: Enzyme Immunoassay
EcR: Ecdysone Receptor
ETH: Ecdysis-triggering Hormone
ETHR: Ecdysis-triggering Hormone Receptor
JH: Juvenile Hormone
OA: Octopamine
TDC2: Tyrosine decarboxylase 2
