# Supplementary figures and images for: Stress-induced reproductive arrest in Drosophila occurs through ETH deficiency-mediated suppression of oogenesis and ovulation
Source: BMC Biol. 2018 Jan 30;16:18. doi: 10.1186/s12915-018-0484-9 (PMC5791332; doi:10.1186/s12915-018-0484-9)

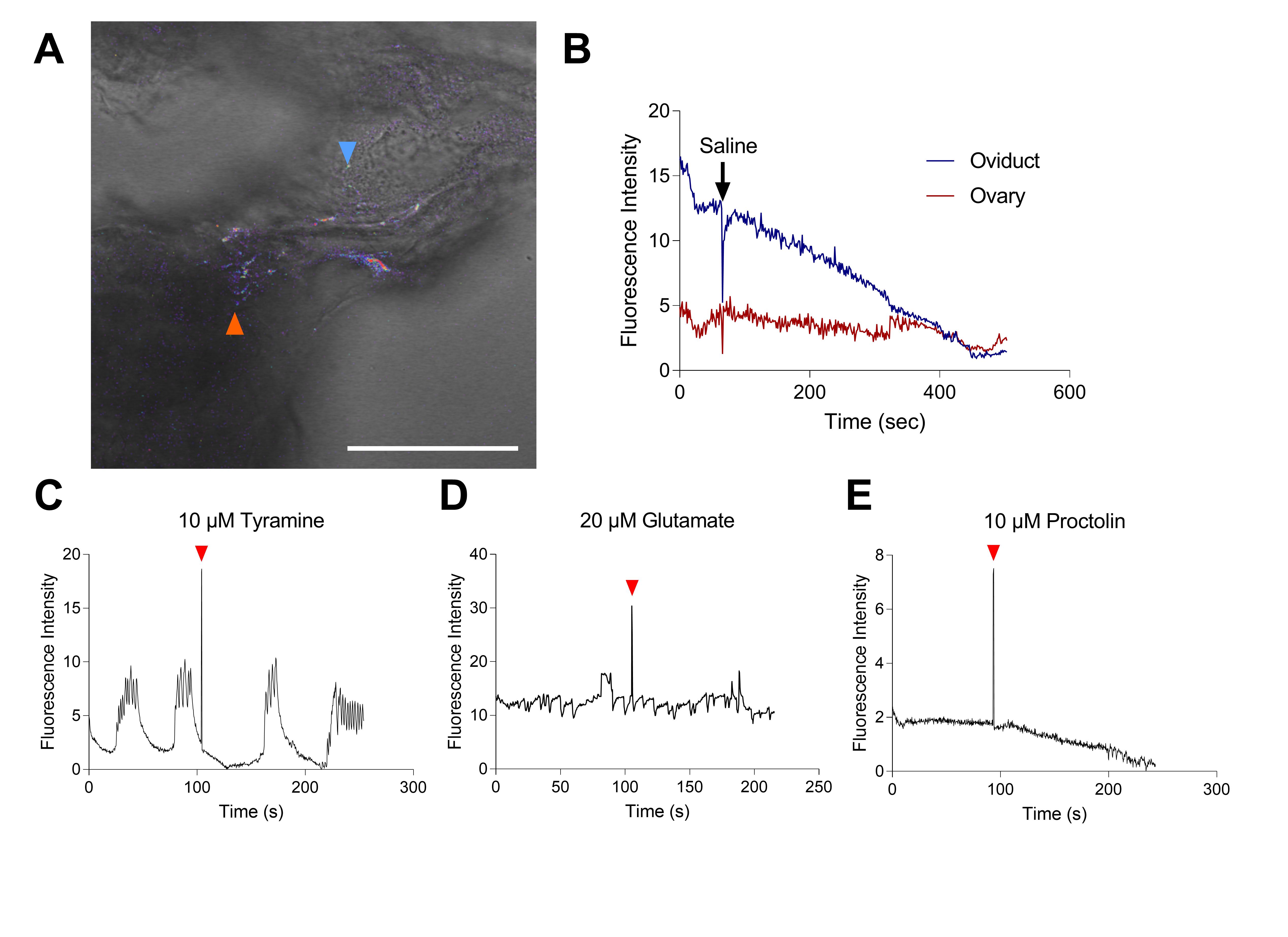

Supplement: Supplementary file 7 — Tyramine, proctolin, and glutamate do not stimulate contractions at the base of the ovary. (A, B) Response to saline in octopaminergic neurons of the (A) oviduct (blue arrowhead) and ovary (orange arrowhead) (scale bar = 100 μm). (B) Nerve terminal fluorescence intensity from (A) over time, before and after treatment. (C–E) Fluorescence intensity in basal ovary epithelium (109-53-Gal4/UAS-GCaMP6S) before and after tyramine (C), glutamate (D), and proctolin (E) at indicated doses (treatment at red arrowheads). (JPG 695 kb) [file 12915_2018_484_MOESM1_ESM.jpg]

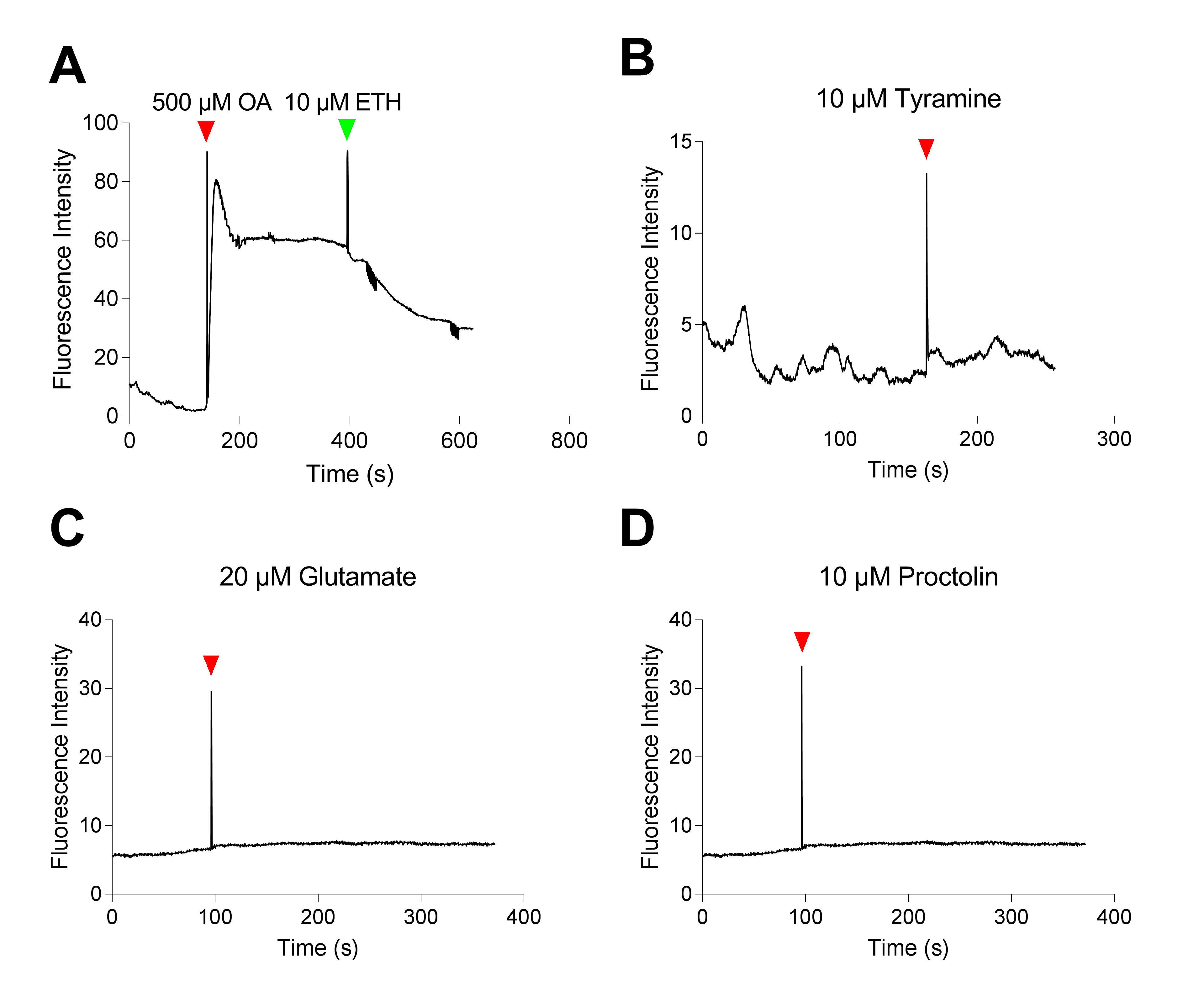

Supplement: Supplementary file 8 — OA, but not tyramine glutamate or proctolin, causes calcium release in the oviduct epithelium. (A) Fluorescence intensity of oviduct epithelium (OAMB-Gal4/UAS-GCaMP6S) after saturating dose of octopamine (500 μM, red arrowhead) and subsequent ETH treatment (10 μM, green arrowhead). (B–D) Oviduct epithelium response to treatment (red arrowheads) with 10 μM tyramine (B), 20 μM glutamate (C), and 10 μM proctolin (D). (JPG 418 kb) [file 12915_2018_484_MOESM8_ESM.jpg]

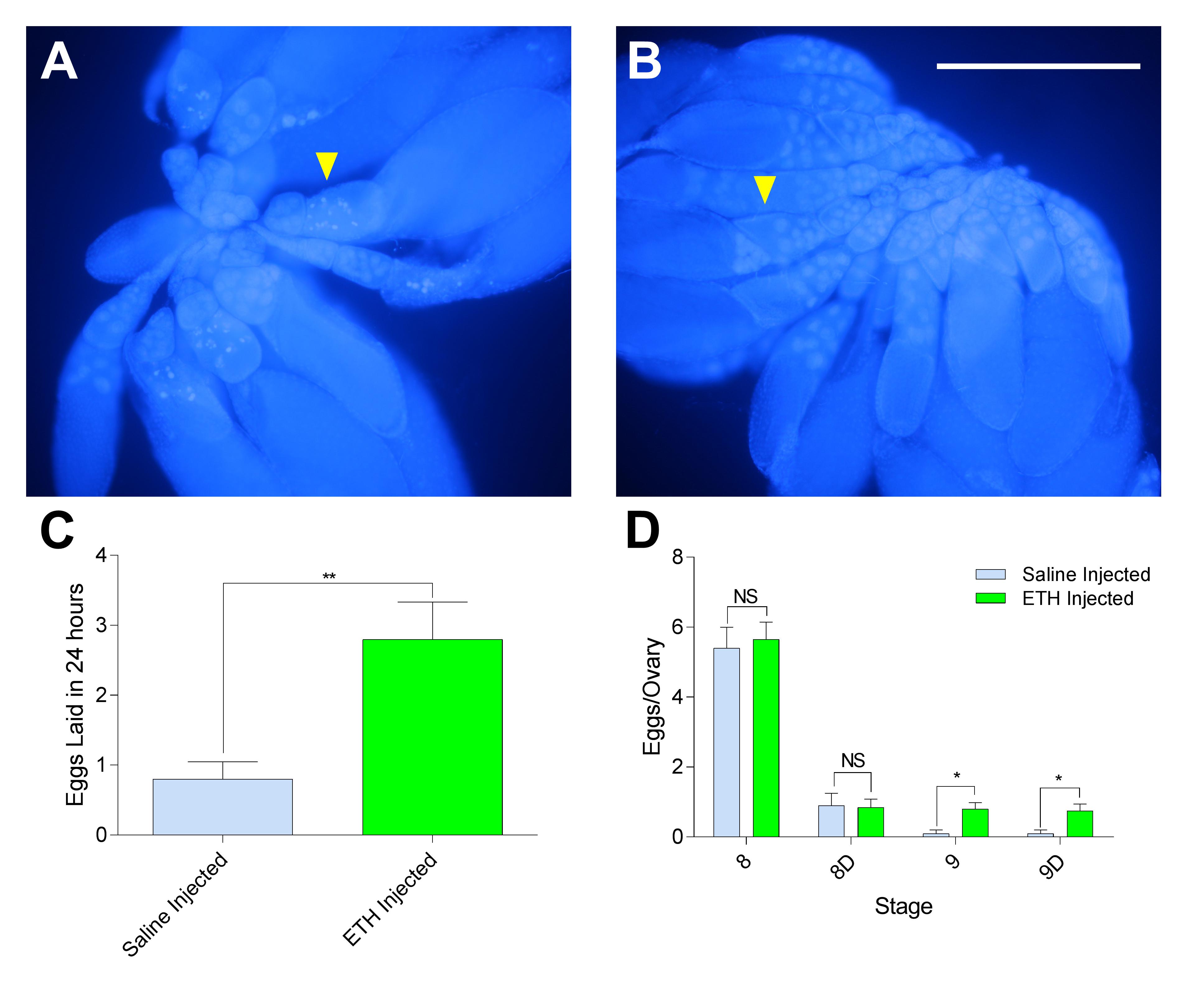

Supplement: Supplementary file 9 — ETH injection rescues oogenesis in heat-stressed females and oogenesis and fecundity in sugar-starved, mated females. (A, B) DAPI-stained ovary from heat-stressed, saline-injected female (A) and one injected with ETH (B), mid-oogenetic oocytes, yellow arrowheads (scale bar = 500 μm). (C) Egg-laying of Canton-S females during 24 h of sugar starvation after injection with saline (n = 23) or ETH (n = 19) just prior. (D) Staging of mid-oogenesis oocytes dissected from ETH- or saline-injected females after 24 h of starvation (n = 20) (biological replicates). Error bars represent SEM. *p < 0.05, **p < 0.01. (JPG 449 kb) [file 12915_2018_484_MOESM9_ESM.jpg]

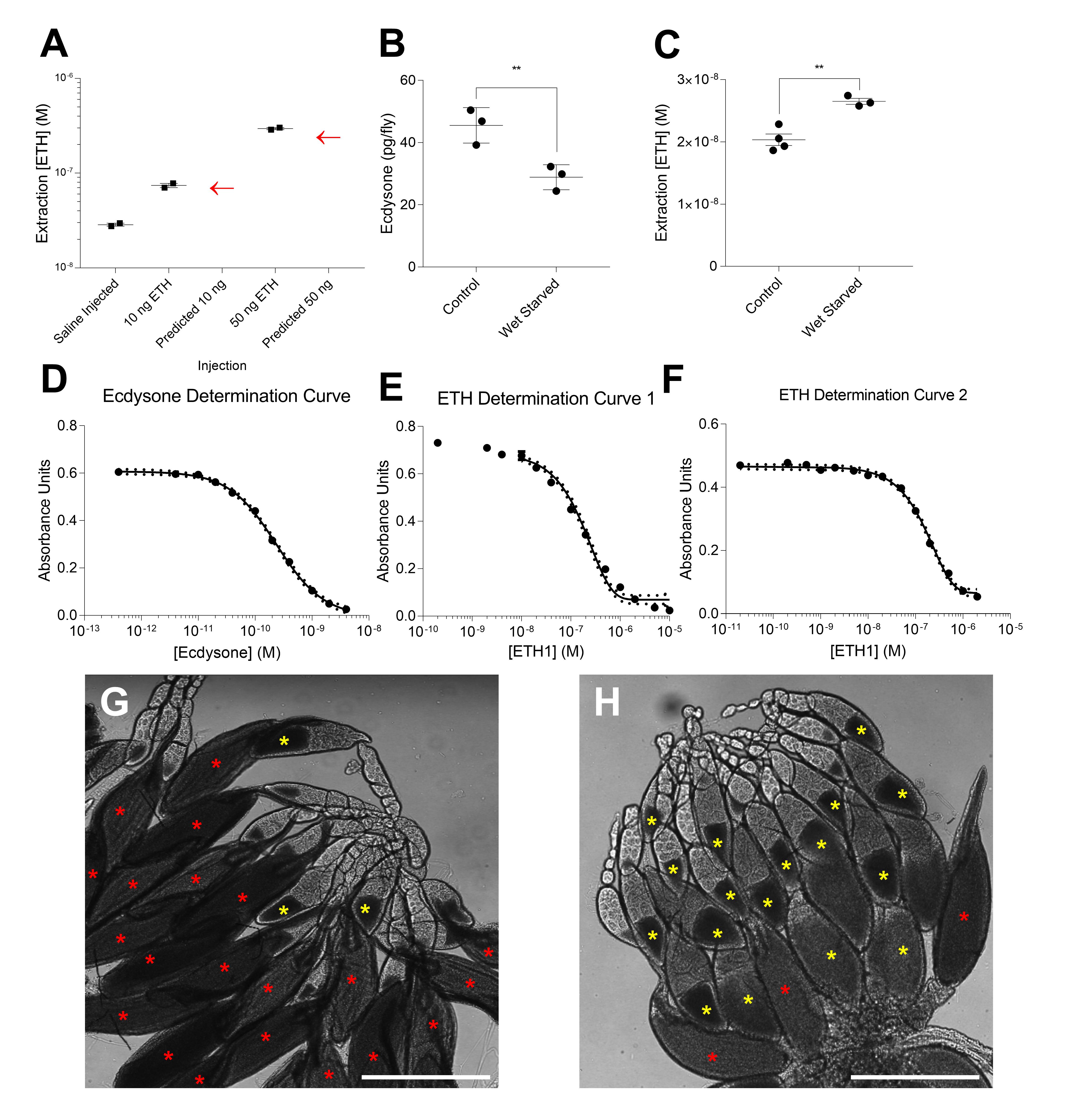

Supplement: Supplementary file 10 — Enzyme immunoassay (EIA) validation, hormonal state change with wet starvation, and genetic rescue of heat stress-induced oogenesis arrest. (A) EIA after injection of saline, 10 ng or 50 ng ETH, along with predicted concentrations based on relative quantity injected (red arrows). Recovery was found to be ~ 2% of the predicted ETH concentration in the hemolymph, assuming a total blood volume of 1 μl. (B, C) Hemolymph ecdysone (B) and ETH (C, ETH determination Curve 1) levels in unstressed and wet starved females. (D–F) EIA standard curves used for quantification of ecdysone (D) and ETH (E, F) levels from Fig. 6a–c, respectively (r square > 0.99). (G, H) Example ovaries from heat-stressed females of the genotype UAS-ßFTZ-F1 (G) and ETH-Gal4;TubulinGal80ts/UAS-ßFTZ-F1 (H). Red arrowheads indicate mature eggs retained, while yellow arrowheads indicate vitellogenic, progressing oocytes (scale bars = 500 μm). (JPG 1.14 mb) [file 12915_2018_484_MOESM10_ESM.jpg]
